# The Effect of Orthographic Neighbourhood and Semantics on Lexical Processing in a Transparent Orthographic Language: A Pupilometry Study

**DOI:** 10.1007/s10936-026-10267-4

**Published:** 2026-06-13

**Authors:** Hazal Artuvan Korkmaz, Özgür Aydın

**Affiliations:** 1 Department of Physiology, Ankara University Medical School, Ankara, Turkey; 2 Ankara University Brain Research Centre, Ankara, Turkey; 3 Neuroscience and Neurotechnology Excellence Joint Application and Research Centre, Ankara, Turkey; 4 Interdisciplinary Neuroscience Program, Ankara University Institute of Health Sciences, Ankara, Turkey; 5 Faculty of Languages, History, and Geography, Ankara University, Ankara, Turkey

**Keywords:** Lexical processing, Visual word processing, Orthographic neighborhood, OLD20, Pseudoword, Pupil dilation

## Abstract

A word’s neighborhood comprises orthographically similar words, yet neighborhood effects in transparent orthographies remain theoretically underspecified. We investigated how lexical status and orthographic distance modulate cognitive effort using pupil dilation during silent reading. Pupillary responses from thirty-four adults were analyzed using a combination of Linear Mixed-Effects models and Generalized Additive Mixed Models across real words (RWs), low-distance pseudowords (PWs), and high-distance nonwords (NWs). Results revealed a temporal hierarchy and a double dissociation between lexical and orthographic processing. Specifically, the lexicality effect (RW > PW) emerged early (1333–2667 ms), reflecting the sustained cognitive load of semantic integration and global activation once lexical access is achieved. In contrast, the orthographic neighborhood effect (PW > NW) appeared at a later stage (2152–2727 ms), manifesting as a delayed peak that reflects the intense lateral inhibition required to suppress competing lexical candidates in PWs. While RWs trigger faster but deeper semantic processing, PWs result in a delayed response reflecting the difficulty of resolving competition among orthographically similar forms. These dynamics refine neighborhood theories by demonstrating that pupillometry can dissociate early-stage semantic activation from late-stage inhibitory control driven by orthographic similarity.

**Supplementary Information:**

The online version contains supplementary material available at 10.1007/s10936-026-10267-4.

## Introduction

### Orthographic Neighborhood Effects in Visual Word Recognition

Orthographic neighbors are words formed by changing one letter (e.g., *cat* → *bat*, *cap*, *car*). Their total number defines neighborhood size or Coltheart’s N (Coltheart et al., 1977). A newer measure, orthographic Levenshtein Distance 20 (OLD20), also considers insertions, deletions, and transpositions across words of different lengths, and is viewed as a more sensitive index since it correlates more strongly with word frequency (Yarkoni et al., 2008). Previous research has shown that orthographic neighborhoods play a key role in visual word recognition (e.g. Forster & Taft, 1994; Grainger et al., 1989; Johnson & Pugh, 1994). It is generally accepted that orthographic neighbors are co-activated during visual word recognition and that the speed of lexical access is influenced by the density of this neighborhood (Andrews, 1997; Perea & Rosa, 2000).

Although several models have been proposed to explain word recognition, evidence remains mixed regarding how lexical and orthographic factors interact. Behavioral studies generally support a facilitative effect of orthographic neighborhood, consistent with interactive-activation and parallel distributed processing models (Andrews, 1997; Sears et al., 1999, 2008). In contrast, some findings reveal inhibitory effects for low-frequency words—specifically those that are less common in the language and thus more susceptible to competition from orthographic neighbors. This pattern aligns more closely with the activation-verification model, which suggests that lexical access involves a serial verification process; in this framework, high-frequency neighbors can interfere with the recognition of a lower-frequency target by creating competition that must be resolved before the target is successfully identified (Huntsman & Lima, 2002; Paap et al., 1982). Consistent with multiple read-out models of neighborhood activation (Grainger & Jacobs, 1996), low distance pseudowords trigger lexical competition mechanisms despite their non-lexical status. Despite extensive behavioral evidence, the physiological correlates of these processes remain underexplored. In particular, there is a notable scarcity of empirical evidence regarding how orthographic proximity influences visual word recognition in orthographically transparent languages.

### Visual Word Recognition in Transparent Orthographies

Extensive research suggests that the transparency of an orthography significantly dictates the cognitive strategies employed during visual word recognition. In two extensive investigations using Spanish stimuli—a language characterized by high orthographic transparency—Carreiras et al. (1993) and Carreiras et al. (1997) found robust evidence for the inhibitory neighborhood frequency effect across multiple identification and categorization paradigms. Carreiras et al. (1993) demonstrated that disyllabic words composed of high-frequency syllables are recognized more slowly than those with low-frequency syllables. They attribute this inhibition to the activation of syllable-based neighbors—words sharing a common syllable in the same position—which compete for lexical access alongside orthographic neighbors. Increased syllable frequency expands the set of activated lexical competitors, thereby delaying target word identification through a process of competitive inhibition. The inhibitory influence of syllable frequency on visual word recognition has also been observed in other Western European languages with relatively less transparent orthographies, such as French (Mathey & Zagar, 2002) and German (Conrad & Jacobs, 2004). In a similar vein, the authors observed in Carreiras et al. (1997) that words with higher frequency neighbors yielded significantly slower response times and higher error rates compared to words without such neighbors. These findings suggest that higher-frequency neighbors act as potent lexical competitors, creating an interference effect that delays the isolation of the target word’s orthographic representation in memory. Furthermore, the study demonstrated that this inhibitory effect persists even in higher-level cognitive tasks. In a semantic categorization paradigm, high-frequency neighbors continued to exert a detrimental influence, particularly when the target words belonged to high-density neighborhoods. Within the framework of the Multiple Read-out Model, these results are interpreted as a consequence of lexical inhibition within an interactive-activation network: the most activated neighbors generate the strongest inhibitory signals, thereby slowing the activation of the target unit until a unique identification criterion is met. Consequently, for readers of transparent orthographies, the competition from more frequent orthographic “neighbors” remains a primary constraint on the efficiency of visual word recognition, independent of the specific semantic or orthographic task demands.

Readers of transparent orthographies appear to rely more heavily on phonological assembly and serial processing compared to readers of opaque orthographies. Ellis and Hooper (2001) demonstrated this distinction by observing that readers of Welsh, a language characterized by a highly transparent orthography, exhibited slower reaction times (RTs) that scaled linearly with word length, alongside a higher frequency of mispronunciation errors for pseudowords. This pattern is further supported by Ziegler et al. (2001), who compared adults reading English—an opaque orthography—with those reading German, which possesses a relatively transparent writing system. Mauti et al. (2023) analyzed lexical decision performance in English and Italian speakers, revealing that while processing speeds were generally comparable, English observers utilized a significantly lower decision criterion—a more lenient “response threshold”—than their Italian counterparts, a finding derived through the application of the Diffusion Model. This lower criterion explains a critical behavioral paradox: English readers were able to maintain reaction times similar to Italian readers despite having lower accuracy.

While current findings provide a robust framework through reaction times and accuracy, there remains a notable lack of pupillometric data regarding the temporal dynamics of lexical competition. Consequently, shifting the focus to pupillary responses allows for a more sensitive measure of the underlying cognitive effort in transparent orthographies.

### Pupillary Sensitivity to Lexical Competition in Visual Word Recognition

Pupillometry measures changes in pupil dilation in response to stimuli or cognitive processes, enabling continuous, temporally detailed data collection (Beatty, 1982; Beatty & Kahneman, 1966; Hess & Polt, 1964). The task-evoked pupil response (TEPR) refers to the small, rapid, and involuntary increase in pupil dilation that occurs as mental effort arouses the sympathetic system during effortful processing. Described by Kahneman (1973) as “the best single index” of cognitive effort, pupillometry has proven to be a sensitive measure across domains such as language, memory, and decision-making. The pupillary response typically initiates as early as 200 ms post-stimulus, though its peak latency and duration are modulated by the cognitive demand of the task (Beatty, 1982).

In linguistic processing, dilation onset and magnitude vary significantly according to word frequency and orthographic similarity, as more taxing lexical searches elicit more sustained responses. High-frequency words engage the lexical route linking orthography to phonology, whereas PWs rely on the slower non-lexical route. Decoding unfamiliar words also recruits additional attentional and motivational resources, increasing cognitive load. Thus, well-known words are decoded faster than unfamiliar ones such as PW and NW (Geller et al., 2016; Güldenoğlu, 2016; Güldenoğlu et al., 2016; Artuvan Korkmaz et al., 2019; Kuchinke et al., 2007; Martin et al., 2006; Miller, 2005; Papesh & Goldinger, 2012). Pupillometry studies showed that high frequency words cause a higher peak pupil dilation than low frequency ones in English (Papesh & Goldinger, 2012) and German (Kuchinke et al., 2007). Geller et al. (2016) reported that high-frequency English words caused earlier pupil dilation than low-frequency words. They also found that dilation increased when words had more lexical competitors, regardless of their frequency. Since PW decoding engages the non-lexical route and demands greater cognitive effort, it is expected to evoke stronger pupil dilation. Indeed, Shechter and Share (2021) observed such an effect across their experiments conducted in Hebrew. In their study, across both adult and child populations, unfamiliar pseudowords consistently elicited greater overall pupil dilation, higher peak dilation, and longer latencies to peak dilation compared to familiar real words. Furthermore, the authors reported a significant familiarity-by-length interaction; whereas real words showed minimal or attenuated length effects, pseudowords exhibited a robust increase in pupillary response as string length increased. These results indicate that the sequential processing required for unfamiliar strings demands significantly more mental resources than the direct memory retrieval utilized for familiar words.

However, empirical validation of this assumption remains scarce, and direct pupillometric evidence distinguishing lexical from non-lexical processing routes has yet to be fully established in the literature. Although previous studies have demonstrated pupil dilation sensitivity to word frequency, it is worth noting that high- and low-frequency words do not represent discrete categories but rather points along a continuous spectrum of lexical familiarity. While a few studies (e.g., Shechter & Share, 2021) have examined pseudowords that are orthographically similar to real words, there is a notable absence of research focusing on strings that bear no orthographic resemblance to existing lexical items. Within this continuum, the extent to which pupil dynamics reflect orthographic distance and lexical accessibility remains an unresolved question in the literature.

### The Present Study and Hypotheses

To investigate the psychophysiological markers of visual word processing, we operationalize three distinct lexical and orthographic conditions: (a) Real Words (RW), representing existing semantic content with low OLD20 values; (b) Pseudowords (PW), which lack semantic content but maintain high orthographic similarity to existing words (low OLD20); and (c) Nonwords (NW), defined by the absence of both semantic content and orthographic proximity (high OLD20). The present study examined how lexicality (RWs vs. PWs) and orthographic neighborhood (PW vs. NW) influence pupil dilation during silent reading in Turkish. This design allowed us to dissociate semantic activation from orthographic neighborhood effects. We formulated the following hypotheses:

- H1 (lexicality effect): Pupil dilation will be greater for RWs than for PWs, reflecting additional cognitive effort associated with semantic activation.
- H2 (orthographic neighborhood effect): PWs (low OLD20) will elicit greater dilation than NWs (high OLD20), due to increased lexical retrieval effort and competition among similar orthographic entries.
- H3 (interaction over time): The time course of pupil dilation will differ across conditions.

Thus, we aimed to capture the dynamic interplay between lexical and orthographic factors in a transparent orthography.

## Method

### Participants

Thirty-four healthy adults, comprising 18 females, between the ages of 18 and 35 (mean = 26.5, SD = 7.77) were included in the study. They were all native speakers of Turkish and had no uncorrected visual impairment, no neurological, neuropsychological, hearing, visual or language impairments. The included participants were right-handed, as assessed by the Turkish version of the “handedness questionnaire” test (Chapman & Chapman, 1987; Nalçaci et al., 2002). A priori power analysis was conducted using the *wp.regression()* function in the *WebPower* R package (Version 0.8.7; Zhang et al., 2023) for fixed effects in linear multiple regression. Assuming *α* = 0.05, power = 0.80, *f*^2^ = 0.35, and two predictors (lexicality and orthographic distance), the required sample size was *N* = 31. To ensure adequate statistical power and account for potential data loss, we recruited 34 participants (see Supplementary Material, Fig. S1). All participants provided written informed consent, and the study protocol was approved by the Local Ethics Committee (Approval No: 2024-000182-2).

### Material

The visual lexical decision paradigm utilized three distinct stimulus categories, all of which consisted of five-letter strings (N = 90). In addition, 20 animal names were included as catch trials to ensure task engagement, resulting in a total of 110 stimuli. These included RWs (N = 30), characterized by meaningful Turkish vocabulary with low OLD20 values (indicating high lexical similarity); PWs (N = 30), which are meaningless strings that also feature low OLD20 values; and NWs (N = 30), distinguished by high OLD20 values (indicating low lexical similarity). The RWs were selected from among the first 2500 words (rank, M = 1023 [199–2383], SD = 582) in the list of the 5000 most frequently used words in Turkish (Aksan et al., 2017). In the process of word selection, particular attention was paid to achieving balance in the averages of Julliand’s “D distribution index” beyond the raw frequency values of the words (raw frequency, M = 11,823 [2077–40233], SD = 9459). This index ranges from 0 to 1, with 1 indicating optimal uniformity of distribution across the corpus. The distribution index of the selected words (M = 0.87 [0.70–0.93], SD = 0.06) indicates that the words appear in a large part of the corpus. In addition to frequency, other factors have been posited as affecting pupil dilation, including the emotional properties and concreteness of words. Consequently, in accordance with the findings from Kapucu et al. (2021)’s study, meticulous attention was devoted to ensuring that the valence, arousal, disgust, and concreteness values were also balanced in the RW selection (valence, M = 6.10 [4.49–7.76], SD = 0.98; arousal, M = 4.94 [3.55–6.49], SD = 0.84; disgust, M = 54.94 [16.80–84.19], SD = 20.33; concreteness, M = 81.45 [30.25–93.91], SD = 13.62).

In order to identify NW and PWs, five-letter words were split into letters using the *strsplit()* function in R, then, permutations of five-letter words were generated using the *permn()* function in the *combinat* package (Chasalow, 2012). Subsequently, the *old20()* function in the *vwr* package (Keuleers & Keuleers, 2013) was applied to the 120 combinations generated to ascertain the OLD20 values for each combination. Orthographic distance was calculated using the lexical database provided by Erten et al. (2014), which comprises 24,414 Turkish stems along with their respective word frequencies. Among these values, excluding the value corresponding to the RW, the combination with the highest OLD20 value was designated as NWs, and the combination with the lowest value was designated as PWs. The nonword group (M = 2.93, SD = 0.11, range: 2.50–3.00) exhibited significantly higher OLD20 values compared to both PWs (M = 1.82, SD = 0.11, range: 1.55–2.00) and RWs (M = 1.78, SD = 0.17, range: 1.50–2.15). A Kruskal–Wallis test confirmed a significant main effect of group (${\chi }^{2}$(2) = 60.36, p < .001), while post-hoc Dunn–Bonferroni comparisons showed that while nonwords differed significantly from the other two groups (p < .001), there was no significant difference between PWs and RWs (p = 1.00).

PWs in the stimuli exhibit a pattern consistent with Turkish syllable structure, while NWs are formed with consonant clusters that do not follow the Turkish syllable structure (see Table S1 in Supplementary Material). It is a well-established fact that Turkish does not generally allow initial consonant clusters or consonant clusters in the final position of a word (Clements & Sezer, 1982; Selen, 1979; van der Hulst & van de Weijer, 1991). In Turkish, initial consonant clusters are observed to a limited extent in loanwords, particularly in written language (e.g., *kral* ‘king’, *tren* ‘train’, *spiker* ‘speaker’, etc.). In spoken language, initial consonant clusters appear to be adapted to the language by inserting a vowel that breaks this cluster according to certain phonological conditions (epenthetic vowel insertion in Yavaş, 1981; feature spreading from the environment in Clements & Sezer, 1982). In the Turkish language, non-initial consonant clusters are also subject to certain restrictions (Ergenç, 1995). The consonant clusters employed in the formation of NWs in this study consist of consonant clusters that are improbable and do not fall within the aforementioned restrictions in Turkish (e.g., *sn, nts, lm, rd, ry, rm, km, yr, pk, mk, vk,* etc.).

To isolate the specific influence of OLD20, we explicitly sought to control for phonotactic probability—often measured through segment and biphone frequencies (Vitevitch & Luce, 1999). Given that all stimuli in our study comprised the same segments, segment frequency was not analyzed; instead, we focused on biphone frequency, which reflects the transitional probability between segments. In a transparent orthography like Turkish, bigram frequency serves as a reliable proxy for biphone frequency, effectively capturing these phonotactic constraints. In line with established Turkish psycholinguistic benchmarks (Erten et al., 2014), we utilized position-specific contiguous bigrams because they represent the most stable statistical units for capturing the local phonotactic constraints of a transparent orthography. While higher-order n-grams like trigrams exist, contiguous bigrams effectively capture the transition probabilities between adjacent segments, which are the most fundamental units of orthographic legality in Turkish. Furthermore, as emphasized by Baayen (2001), trigram distributions are characterized by a substantially higher density of rare events (hapax legomena) compared to bigrams, which leads to “data sparsity” issues that can introduce instability into statistical modeling. In a corpus of Turkish, many potential trigrams yield zero or near-zero frequencies, potentially resulting in unreliable estimates for five-letter strings.

Following this rationale, bigram frequencies were calculated using the *strngrams* package to determine the phonotactic legality of the stimuli. Specifically, a position-specific bigram frequency database was first generated from a Turkish lexicon using the *get_ngram_frequencies* function. Subsequently, the mean token-based bigram frequencies for each stimulus were computed using the *ngram_frequency* function, providing a metric for the phonotactic probability of the five-letter strings. We incorporated position-specific constraints to account for the positional probability of letter sequences within the specific rules of the Turkish language. This method is critical for identifying nonwords that contain legal bigrams in prohibited positions; for instance, while “sn” is frequent medially (e.g., *esnaf* ‘tradesman’, *esnek* ‘flexible’, *nesne* ‘object’ etc.), it is prohibited word-initially. By using position-specific calculations, stimuli such as *sneib* correctly receive a frequency of zero, reflecting their status as phonotactically invalid strings. The analysis revealed a significant main effect of group on bigram values (Kruskal–Wallis ${\chi }^{2}(2)$ = 55.92, p < .001). To account for potential confounds between orthographic distance and phonological factors, bigram frequency was included as a covariate in the models to statistically control for the effects of phonotactic legality and pronounceability. Post-hoc comparisons using the Dunn–Bonferroni method showed that NWs (M = 6.24, SD = 1.10, range: 4.11–8.01) had significantly lower bigram values compared to both PWs (M = 8.63, SD = 0.60, range: 7.15–9.79, p < . 001) and RWs (M = 8.84, SD = 0.73, range: 6.80–9.84, p < .001). In contrast, no statistically significant difference in bigram values was observed between the PW and RW conditions (p = .877, for k-means clustering analysis, see Fig. S2 in Supplementary Material).

Furthermore, the experiment incorporated 20 animal names, each comprising five letters, to be utilized in the task. The experimental stimuli, comprising RWs, NWs, and PWs, as well as animal names, were all created as lemmas, with no grammatical morphemes employed.

### Procedure

The laboratory environment was dimly lit and isolated from external auditory stimuli. Participants were positioned in front of a 22-inch LED monitor with a resolution of 1920 × 1080 pixels and a refresh rate of 60 Hz. To ensure accurate calibration, and to maintain a fixed eye-to-monitor distance of 60 cm, participants’ head movements were minimized by the presence of a chin rest.

Prior to the initiation of the recording procedure, the subject’s dominant eye was ascertained; however, recordings were obtained from both eyes. It was observed that all participants exhibited right eye dominance. The participants were asked to silently read the given words and press the space bar on the keyboard as soon as they read a word belonging to the animal name category. Simultaneously, pupil dilations were measured at a 1000 Hz sampling rate using an SR Research EyeLink 1000 Plus system. The Experiment Builder (SR Research Ltd., 2024) was used to create and present the stimuli. Each trial started with a calibration using the 9-point calibration task. Stimuli were presented from the center of the screen in a mixed order. The text was black (Courier New, horizontal 4.17°) on a grey background (R: 153, G: 153, B: 153, Alpha: 255). In order to ensure that participants were prepared for the experimental procedure, the session began with a practice run.

**Fig. 1:**
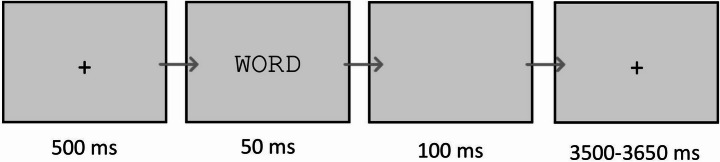
Outline of the experimental design

A total of 110 trials were conducted for the study. Each trial began with a 500 ms fixation cue (a plus sign) in the center of the screen, followed by a 50 ms presentation of a stimulus. This brief duration was strategically selected to target the automaticity of early visual-orthographic activation (Pollatsek et al., 2006) while preventing strategic phonological recoding. This was followed by a 100 ms blank screen, after which a fixation cue appeared in the center for 3500–3650 ms, allowing the pupil size to return to its resting position. Participants were asked to indicate whether the word they had read referred to an animal. They responded by pressing a key. Each trial was presented pseudo-randomly (see Fig. 1). Experiment sessions lasted approximately 45 min, including practice, calibration and breaks. At the end of the experiment, participants were asked to look at a fixation cue for 6300 ms to measure their resting pupil dilation.

### Preprocessing

The preprocessing steps were performed in accordance with the general practices described in the literature (Mathôt et al., 2018). The raw baseline pupil data were obtained using *DataViewer* software (SR Research Ltd., 2025) and then preprocessed using the *PupilPre* package (Kyröläinen et al., 2019) in R. The *pupil_deblink()* function in the *PupilPre* package was used to remove blinking data from the raw pupil data. The utilisation of this function effectively eliminated the blinking effect for the duration between 100 ms prior to the onset of blinking and 100 ms subsequent to the cessation of blinking. Subsequently, the *pupil_artifact()* function was applied using the method proposed by Kret and Sjak-Shie (2019) to remove speed outliers. Blinking was observed in 71.60% of the events recorded from all participants (N = 2556). On average, 6.52% (SD = 10.19) of the events were artefacts. Subsequently, a Hanning filter was applied to correct the raw pupil data prior to linear spline interpolation. Subsequently, linear interpolation was employed to impute the missing values. For each trial, the baseline pupil dilation was calculated by taking the average of the pupil dilation in the – 300 to 0 ms time interval before the onset of the stimuli. Subsequently, a standard baseline subtraction was performed for each trial to facilitate comparison of relative changes in pupil dilation. The data were then down sampled to 50 Hz at the end of the preprocessing stage. It should be noted that the data from the right eye were the only data that were preprocessed and analyzed. Trials in which excessive blinking was observed, exhibiting artifacts exceeding 50% during the baseline period, or during the subsequent period (0–3000 ms), were excluded from the dataset. As a result of this data extraction process, the total missing data was calculated as 3.53%.

### Statistical Analysis

In this study, the initial phase of analysis entailed the examination of several conventional static measurements of pupil response curves. The objective of this analysis was to determine the general appearance of the pupil response. However, when the objective was to obtain findings related to the temporal formation of an effect, such traditional analyses were insufficient to show the pupil response signal developing over time (van Rij et al., 2019). Consequently, in our study, in addition to the conventional approach for analyzing pupil measurement data, we employed generalized additive mixed models (GAMM) analysis to perform a dynamic analysis of the pupil response.

In our models, Lexicality denotes the factor where NWs are excluded and only RWs and PWs are compared; meanwhile, Orthographic Neighborhood refers to the factor where RWs are excluded and PWs and NWs are compared. Therefore, the Lexicality factor was defined to isolate and examine semantic effects while controlling for OLD20. Conversely, the Orthographic Neighborhood factor was defined to isolate the influence of orthographic distance while controlling for semantic effects.

In the traditional analysis, common static measurements of pupil response curves were analyzed: mean pupil diameter, peak pupil amplitude, and the latency of the peak pupil dilation. The mean pupil diameter and the peak pupil amplitude were defined as the average pupil dilation relative to the baseline and the largest value relative to the baseline, respectively. Conversely, the latency of the peak pupil dilation was defined as the time interval between the onset of the stimulus and the time of peak pupil dilation. To identify the most sensitive time window for lexical processing, we conducted an exploratory moving window analysis using consecutive 100 ms windows with 30 ms increments. Based on the results of this exploratory approach, these static indices were calculated within the 600–1000 ms post-stimulus interval. The raw or standardized values of these three measures were analyzed using Linear Mixed-Effects (LME) regression models with the *lme4* package (Bates et al., 2015) and *lmerTest* package in R. P-values for linear mixed models were obtained using the Satterthwaite approach (Satterthwaite, 1946). LME models were calculated with fixed effects for Lexicality (RW, PW) and Orthographic Neighborhood (low, high OLD20, i.e., PW and NW), with log-transformed bigram frequency included as a covariate. Random effect structures were specified for all models, ranging from subject-only random intercepts (for mean pupil dilation) to crossed random intercepts for subjects and items (for peak latency), and by-subject random slopes for the Lexicality effect (for peak amplitude) (Baayen et al., 2008). In order to circumvent the possibility of bias, sum coding was utilized for categorical variables (i.e., − 0.5 vs. 0.5) The *buildmer()* function (buildmer package, Voeten, 2021) was utilized for the purpose of model comparisons (see Table S4). Furthermore, Orthographic Neighborhood (OLD20) was treated as a categorical factor in the sum-coding scheme, while its continuous distribution was found to be normal, requiring no further normalization.

Secondly, the temporal course of pupil dilation was analyzed. GAMMs (Hastie & Tibshirani, 1990; Wood, 2017) were utilized to analyze the temporal course of pupil dilation because it had been demonstrated to be a suitable modelling technique for nonlinear (as well as linear) trends that are typical in pupil measurements over time (see van Rij et al., 2019). Furthermore, GAMMs facilitate the analysis of autocorrelation, which refers to the serial dependence present within time series data (Baayen et al., 2008). In this study, pupil dilation was analyzed for 3000 ms after the onset of the word. In our model, we included the AR-1 correlation parameter ρ = 0.84 to account for autocorrelation. Furthermore, given its classification as a mixed model, it permits the incorporation of random factors into the model, similar to LME. In this study, the GAMM incorporated random intercepts for both participants and items. The model incorporated two factors: Lexicality (RW, PW) and Orthographic Neighborhood (low, high OLD20). The incorporation of two-way interactions between time and variables (Lexicality, Orthographic Neighborhood) was facilitated by the smoothing function of GAMMs, specifically using *s(Time, by* = *Condition)* and binary difference smooths for specific contrasts. Additionally, a two-dimensional tensor product smooth *te(Time, Bigram.log)* was included in all models to control for the non-linear effect of bigram frequency over time.

The graphing procedures were performed using the R statistical environment, version 4.3.2 (R Core Team, 2024), with the *mgcv* package version 1.9.1 (Wood, 2017), and the *itsadug* package, version 2.4.1 (van Rij et al., 2017). Furthermore, the appraise function in the *gratia* package was utilized to verify the number of basis functions for predictors and interactions (Simpson, 2025). The summaries of the final model are presented in the Supplementary Material, while the visualizations of the results are presented in the Results section. In accordance with the methodology outlined by Wieling (2018), the differences were analyzed through the implementation of binary difference smoothing. The precise details of these findings are provided in the Results section. In order to address the role of the OLD20 value on the pupil response, two-dimensional tensor products (*te(Time, OLD.value, by* = *Condition*) and *te(OLD.value, Bigram.log, by* = *Condition)*) were utilized to allow for the modeling of nonlinear interactions between predictors such as time, Orthographic Neighborhood to allow for the modeling of nonlinear interactions between predictors such as time, OLD20 values, bigram frequency, and pupil dilation.

## Results

### Static Analysis: Global Effects on Pupil Dilation

As demonstrated in Fig. S3 (Supplementary Material), a peak is observed at approximately 800–850 ms across conditions. The LME results (Table S5 in Supplementary Material) clarify the underlying drivers of this activity: Orthographic Neighborhood (OLD20) had a significant impact on both the mean baseline-corrected pupil dilation (ß = − 0.11, p < .05; RW: M = 0.36, SD = 0.71; PW: M = 0.34, SD = 0.69; NW: M = 0.23, SD = 0.68) and peak amplitude (ß = − 0.11, p < .05; RW: M = 0.60, SD = 0.74; PW: 0.57, SD = 0.72; NW: M = 0.46, SD = 0.71). This indicates that strings with higher lexical similarity (low OLD20) recruit more intensive cognitive resources. Notably, while orthography drove the magnitude of the response, Lexicality was the sole significant predictor of peak latency (ß = − 28.62, p < .001), with RWs reaching their processing peak earlier than PWs and NWs (RW: M = 822, SD = 155; PW: M = 851, SD = 155, NW: M = 848, SD = 155).

However, these LME indices are essentially static measures that aggregate pupillary activity, potentially masking the non-linear temporal dynamics of the cognitive process. To overcome this limitation, we subsequently conducted Generalized Additive Mixed Models (GAMM). While GAMM shares the robust hierarchical structure of LME by accounting for random effects, it extends the analysis by incorporating smooth terms to model how these lexical and orthographic effects unfold and fluctuate over continuous time. This transition from static to time-resolved modeling allows for a more granular inspection of when exactly these experimental manipulations begin to modulate the pupillary signal.

### Temporal Dynamics of Pupil Dilation

#### The Lexicality Effect: Comparison of Real Words and Pseudowords

**Fig. 2:**
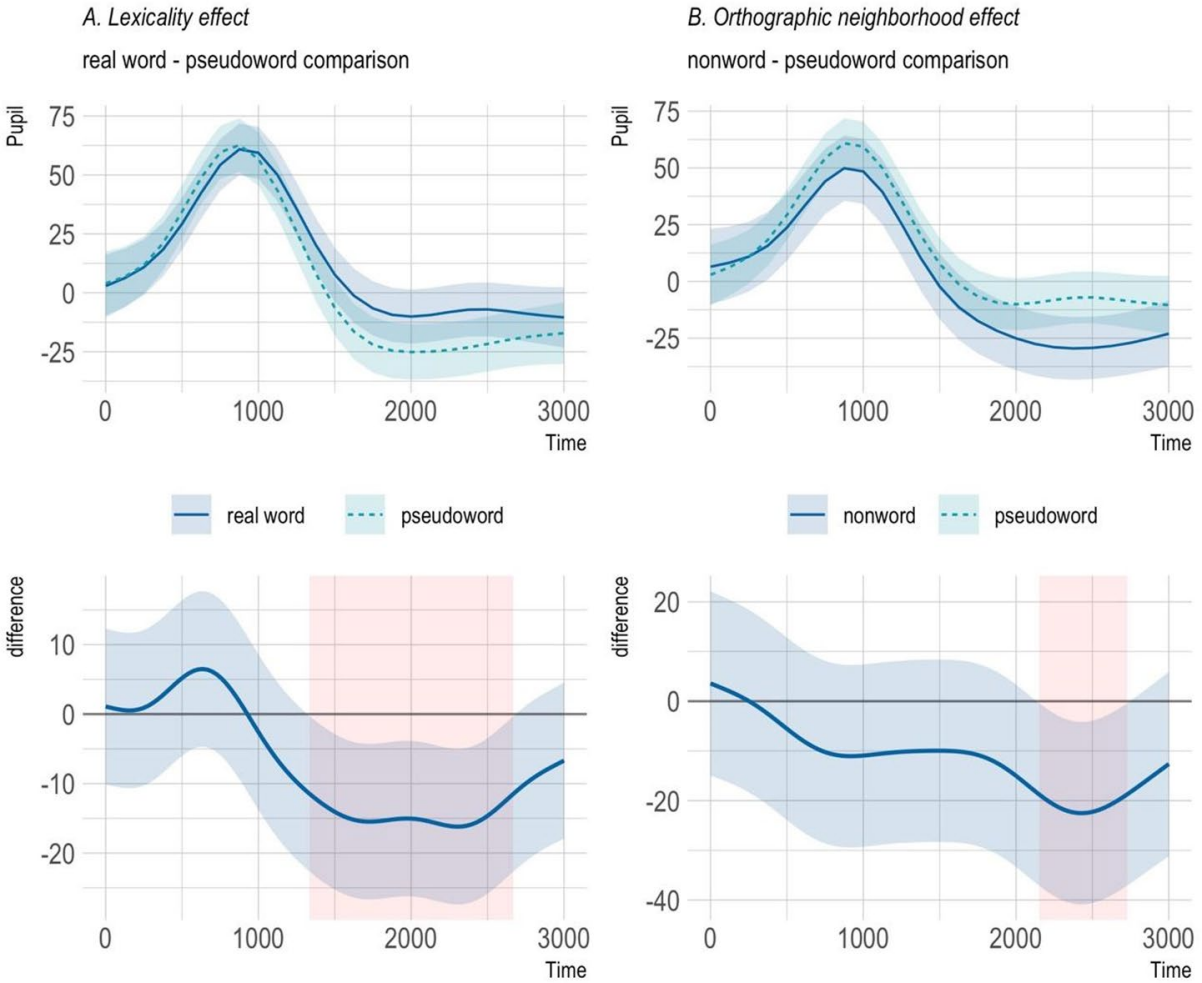
GAMM-smoothed pupil dilation time courses. **A** Lexicality effect (real word vs. pseudoword comparison). **B** Orthographic neighborhood effect (nonword vs. pseudoword comparison). Upper panels show baseline-corrected pupil diameters, while lower panels show the estimated difference curves between conditions. Red shaded areas indicate significant time windows (p < .05) where the difference curve significantly deviates from zero. Random effects are excluded for visualization

The results of the final GAMM, with its parametric and smooth components reported separately, are presented in Table S6 (in Supplementary Material). The findings of the model suggest that it is non-linear. We used the model output’s (i.e., Table S6) smooth and difference graphs to see the time interval in which the regression lines differed for each level of the prediction variable. As demonstrated in Fig. 2 (Panel A), a clear distinction in pupil dilation patterns between RWs and PWs was observed, with significant differences occurring between 1333 and 2667 ms.

The evaluation of these differences was conducted utilizing a new model based on a binary difference smoothing model (Wieling, 2018). In this new model, the two regression lines for the two conditions (RW and PWs) at the low OLD20 value in the first model have been replaced with a reference curve. A full result of this model can be found in Table S6 (see Supplementary Material), while a model summary, highlighting the most essential information, is presented in Table 1. Bigram frequency was included as a covariate in the models to serve as a proxy for phonotactic legality and pronounceability. This covariate significantly influenced the Orthographic Neighborhood model (p = .025) but did not reach significance in the Lexicality model (p = .127). These GAMM results align with the cluster analysis (see Fig. S2 in Supplementary Material), which demonstrated that RWs and PWs occupied overlapping clusters, while NWs formed distinct, separate groups. Furthermore, these findings are consistent with the Kruskal–Wallis analysis of the stimulus metrics: while PWs and RWs showed no significant difference in bigram frequency, NWs differed significantly from both categories (see Table S3 Supplementary Material). As seen in Table 1, Lexicality effect, i.e., the interaction between time and lexical status (*s*(Time):IsRealWord) was significant (edf = 7.70, F = 2.94, p < .01), suggesting that the time course of pupil dilation differed significantly between RW and PWs. This lexicality effect manifested as a larger and more sustained pupillary response for RWs, reflecting the increased cognitive effort required for lexical retrieval and semantic integration.

**Table 1:** Summary of GAMM results for experimental effects and bigram frequency control across two separate models

Comparison (difference smooth)	edf	F	p	Significant time window (ms)
Lexicality (RW vs. PW)	8.36	3.51	< .001	1333–2667
Orthographic neighborhood (NW vs. PW)	5.23	2.34	0.034	2152–2727
Bigram frequency (control)				
In lexicality model	3.37	1.8	0.127	–
In neighborhood model	2.02	3.68	0.025	–

*edf* effective degrees of freedom

This table provides a summary of the binary difference smooths and control variables from two separate GAMM models (see Supplementary Material, Tables S7 and S8). Significant time windows indicate periods where the difference curves significantly deviate from zero (p < .001). Full statistical summaries for both models, including parametric coefficients and random effect structures, are provided in the Supplementary Material

#### Orthographic Neighborhood Effect: Comparison of Nonwords and Pseudowords

When the smooth and difference graphs of the model output (i.e., Table S6 in Supplementary Material) are plotted (see Fig. 2 Panel B), a clear distinction is observed between NWs and PWs; specifically, PWs elicited a significantly larger pupil amplitude compared to NWs, reflecting the increased cognitive effort involved in processing items with high OLD20 values that closely resemble real lexical entries..

In terms of temporal dynamics, as demonstrated in Fig. 2 (Panel B), the effect of orthographic neighborhood density manifests between 2152 and 2727 ms. Notably, this orthographic effect emerges subsequent to the time window in which the lexicality effect is observed. This pattern suggests that OLD20 modulates processing at a later stage, following the initial engagement of semantic and lexical systems.

In order to evaluate these differences, a new model was developed, based on the binary difference smooth model, as was previously employed. In this new model, a reference curve was created for two conditions with low and high OLD20 values, with the RWs excluded from the first model. The complete results for this model are detailed in Table S8, with the *IsHighOLD* reference curve (Orthographic Neighborhood effect) and Bigram effects specifically presented in Table 1. Orthographic Neighborhood effect, i.e., the interaction between time and *IsHighOLD* was significant (*s*(Time):IsHighOLD, *p* < .05), suggesting that high-OLD20 NWs elicited a different pupillary response pattern over time compared to low-OLD20 PWs. These results suggest that orthographic neighborhood proximity (as captured by OLD20) modulates cognitive load, thereby influencing the temporal dynamics of pupil dilation and reflecting the specific processing demands during PW recognition.

#### Three-Way Interaction of OLD20 Value, Time, and Pupil Response

In the preceding analysis, the effect of orthographic distance was examined in isolation from the semantic content. This prompts the following question: The central question guiding this study is whether there is a difference in orthographic distance between cases where there is semantic content (i.e., RWs) and cases where there is no semantic content (i.e., PWs). As a complementary analysis, we generated a tensor product model and a contour plot of this model to examine the effect of OLD20 value on pupil dilation in both RWs and PWs. Specifically, we explored the three-way interaction between time, OLD20, and pupil dilation, moderated by the RW, PW and NW. This allowed us to assess how lexical similarity (indexed by OLD20) influences pupillary responses under different lexical conditions, particularly when OLD20 is low—i.e., when the letter combination probability is high. A summary of this model can be found in Table S9 in the Supplementary Material.

**Fig. 3:**
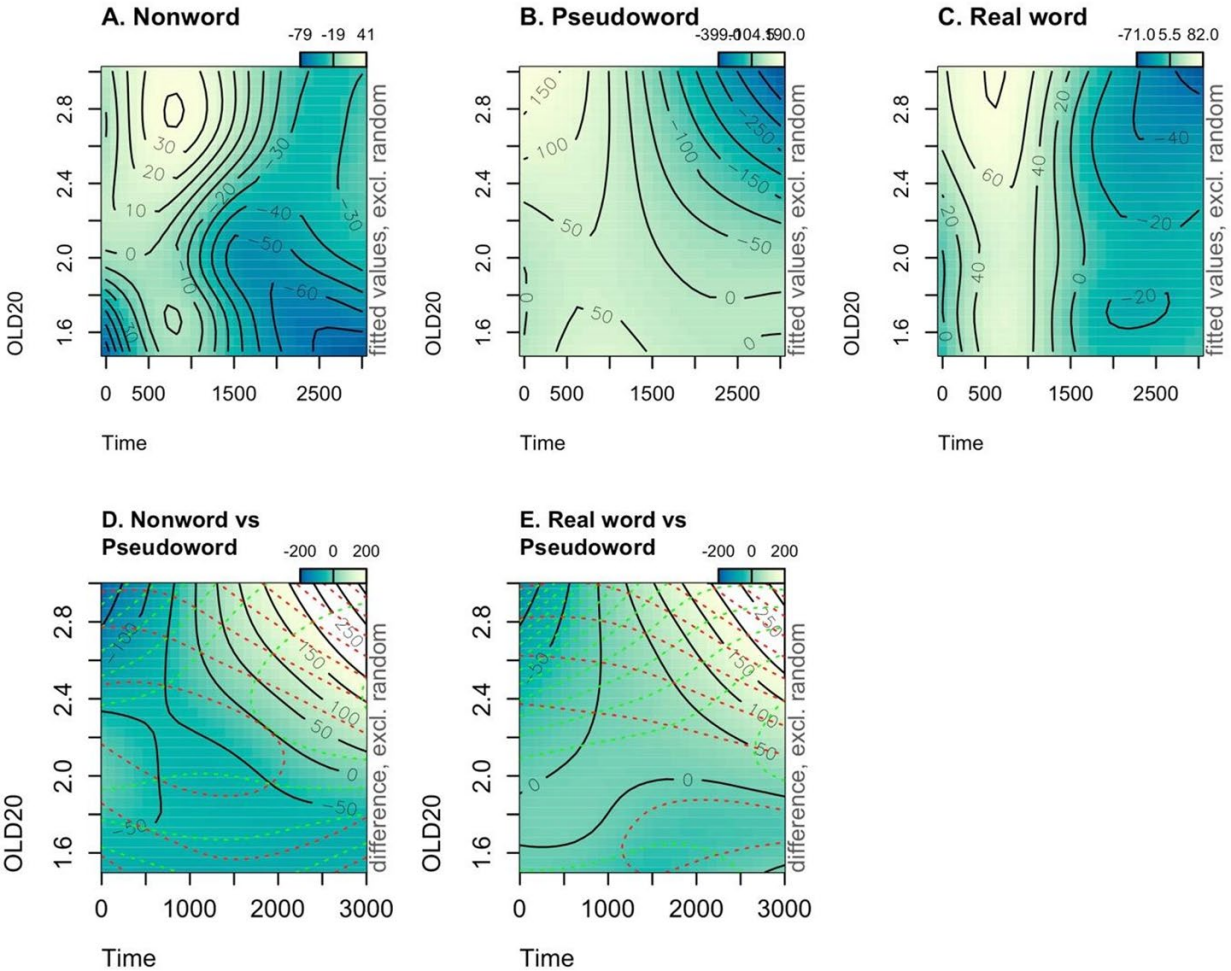
Contour plots and difference surfaces of the tensor product interaction between Time and OLD20 for each lexical condition. Panels **A**, **B**, and **C** display the fitted pupillary surfaces for NWs, PWs, and RWs, respectively. The x-axis represents Time (ms) and the y-axis represents orthographic distance (OLD20). Yellow/lighter colors indicate higher pupil dilation (increased cognitive effort), while blue/darker colors signify constriction or return to baseline. Solid black contour lines represent levels of predicted pupil size. Panels **D** and **E** show the statistical difference surfaces (NW minus PW and RW minus PW). In these difference plots, blue regions indicate lower pupil dilation for the first condition compared to the second. The red-dashed lines and green-dotted shaded areas highlight the specific spatio-temporal regions where the differences between conditions are statistically significant

The Generalized Additive Mixed Model (GAMM) results confirm that the interaction between Time and OLD20 is a significant predictor of pupillary responses across all lexical conditions (p < .001 for all tensor products, see Table S9). Figure 3 was generated based on the GAMM results detailed in Table S9. In Panels A–C, the color gradient serves as a proxy for cognitive load: yellowish peaks indicate maximum resource allocation, while teal and blue valleys represent lower levels of effort. The contour lines connect points of equal pupillary change, providing a topographical view of processing intensity. As illustrated in Panels A and B, both NWs and PWs elicit the greatest cognitive effort when orthographic distance is low (closer proximity to real words), particularly between 500 and 1500 ms. This is evidenced by the prominent yellow clusters at the lower end of the y-axis. As OLD20 increases—indicating letter strings that are more orthographically “distant” from other words—dilation notably decreases, suggesting that strings with low lexical similarity are processed with less cognitive demand. In contrast, Panel C (RWs) reveals a more vertically distributed and stable dilation pattern, indicating that for existing lexical items, orthographic proximity does not modulate processing effort as intensely as it does for unfamiliar strings.

In the difference plots (Panels D and E), green-dotted regions and red-dashed lines demarcate the specific spatio-temporal areas where the interaction between time and OLD20 differs significantly across lexical conditions. Panel D confirms a significant divergence in processing effort between NWs and PWs. This aligns with our previous analysis showing distinct pupillary responses in the later processing stages. Panel E provides critical new insight by directly comparing RWs and PWs through an orthographic lens. The results reveal that when orthographic similarity is high (low OLD20), PWs elicit a substantially more robust and prolonged pupillary response compared to RWs. This demonstrates that the “lexicality effect” is heavily modulated by orthographic distance; cognitive effort for PWs increases as they become orthographically closer to RWs, a modulation that is largely absent in actual lexical items.

## Discussion

This study investigated how lexical status and orthographic similarity modulate pupil dilation during silent reading in a transparent orthographic system. By using both static pupil metrics and time-resolved GAMM modelling, we revealed that cognitive effort during word processing unfolds in distinct temporal phases. Based on our hypotheses (H1–H3), we differentiated between semantic activation effects arising from real-word processing and orthographic neighborhood effects arising from graded similarity among RWs, PWs and NWs.

### Lexicality Influences Pupil Dilation

Consistent with H1, RWs elicited greater pupil dilation than PWs between 1333 and 2667 ms, indicating that late-stage semantic processing imposes an additional cognitive load. The significant difference between RW and PWs confirms a lexicality-driven semantic effort, validating H1. According to the global lexical activity model, when a PW is presented, the level of global lexico-semantic activity is higher because there are only lexical neighbors, but no semantic neighbors; thus, the suppressive effect of the neighbors is reduced. This activity results in an increased N400 amplitude in the EEG (Meade et al., 2018, 2019, 2021).

According to the E-Z Reader model (Pollatsek et al., 2006), the completion of the initial visual processing stage typically requires approximately 50 ms. This duration is grounded in the ‘eye-mind latency,’ which accounts for the time required for visual information to travel from the retina to the striate cortex (e.g., Clark et al., 1994). The methodological differences between the present study and that of Shechter and Share (2021) provide a plausible explanation for the divergent pupillometric findings. In the study conducted by Shechter and Share (2021), the orthographic representation of the stimulus remained on the screen throughout the lexical processing phase (up to 3300 ms or until a response was made). Furthermore, their experiments incorporated an oral reading task, which inherently imposes a higher cognitive load on PWs compared to RWs. In the case of PWs, the absence of a pre-existing lexical entry forces the parser to engage in iterative phonological recoding—a serial process of grapheme-to-phoneme mapping. This labor-intensive decoding process likely accounts for the significant pupil dilation observed in their study as a marker of cumulative cognitive effort. In contrast, the 50 ms stimulus duration employed in our study targets the automaticity of early visual-orthographic activation before such resource-heavy, strategic phonological recoding can fully manifest.

The temporal dynamics of our findings provide critical insights into the distinct nature of early-stage lexical access and late-stage semantic processing. While the GAMM results highlighted a late-stage semantic load, the LME analysis showed that lexicality also impacted the initial phase of processing. LME results showed that lexicality significantly impacted peak latency, with PWs exhibiting a delayed peak pupillary response that points to an early-stage processing difficulty in comparison to RWs. While RWs elicit an earlier peak due to rapid, automatic access to the mental lexicon, the significantly greater dilation observed for RWs in the GAMM analysis (1333–2667 ms) indicates that once lexical access is achieved, the subsequent semantic integration and global activation impose a more substantial and sustained cognitive load than the unsuccessful lexical search associated with PWs. Thus, our results demonstrate a double dissociation: in the early stage (600–1000 ms), RWs show shorter peak latencies due to rapid lexical access and pre-existing mental representations. Conversely, in the late stage (1333–2667 ms), RWs elicit higher amplitudes, reflecting sustained post-lexical semantic processing and network activation. While PWs are quickly discarded as non-lexical, RWs require deeper cognitive engagement for meaning integration.

### Orthographic Neighborhood Influences Pupil Dilation

Consistent with the directional prediction in H2, PWs with low OLD20 exhibited greater dilation than NWs with high-OLD20. This suggests that high similarity to RW forms in PWs triggers stronger lexical competition, demanding inhibitory control to suppress multiple activated neighbours. One explanation could be that the probability of orthographic co-occurrence increases with lower OLD20 values, indicating a higher global lexico-semantic efficiency (Grainger & Jacobs, 1996; Yarkoni et al., 2008). In contrast, high-OLD20 NWs are too dissimilar to engage lexical memory networks, resulting in reduced cognitive effort and lower dilation. These findings indicate that orthographic familiarity—not unfamiliarity—is the driver of cognitive load, meaning H2 is empirically supported in terms of competition-based effort. This is a theoretically meaningful finding that strengthens inhibitory neighborhood models (Grainger et al., 1989; Sears et al., 1995). Meade et al. (2018) hypothesized that when a word is presented, the lexical neighbors of the word are also identified and suppressed, probably through lateral inhibition. PWs with low OLD20 values have many close real-word neighbors, thereby increasing the cognitive load and intensifying the processing effort, as the brain must sift through similar lexical candidates before identifying the PWs.

Our findings align with research in transparent orthographies, where inhibitory neighborhood effects are prominent. The increased pupil amplitude for high-OLD20 PWs mirrors the competitive inhibition observed in Spanish (Carreiras et al., 1993, 1997), where high-frequency neighbors delay target identification. Given that readers of transparent systems, such as German or Welsh, rely heavily on phonological assembly (Ellis & Hooper, 2001; Ziegler et al., 2001), the presence of numerous orthographic neighbors likely intensifies the cognitive demand of resolving lexical competition. Furthermore, the late emergence of this effect (2152–2727 ms) is consistent with the higher decision criteria typically employed in transparent orthographies like Italian (Mauti et al., 2023).

### Distinct Time Courses for Lexical Versus Orthographic Processing

Our findings indicate that the pupillary response reflects not only the magnitude of cognitive load but also the specific cognitive layer at which this load occurs. Analysis of the GAMM difference curves reveals a clear temporal hierarchy between lexicality and orthographic neighborhood effects. As the primary stage of this hierarchy, the distinction between RWs and PWs becomes significant at exactly 1333 ms, confirming that the process of verifying a stimulus’s semantic representation within the mental lexicon occurs during the relatively early stages of processing.

In contrast, the comparison between PWs and NWs with varying neighborhood densities only emerges as significant at 2152 ms, indicating a distinct temporal lag. This delay can be elucidated through two integrated theoretical perspectives. Firstly, this process can be grounded in lateral inhibition models within the framework of the Interactive Activation Model. As emphasized by Grainger and Jacobs (1996), high neighborhood density initiates a mutual suppression process among similar candidates in the mental lexicon. When the stimulus is a PW, no single candidate reaches a “winner-takes-all” status, leading to prolonged competition (Sears et al., 1995). The late peak observed at 2152 ms in our data aligns with the “late-stage inhibitory control” proposed by Meade et al. (2018). Secondly, according to the Dual-Route Cascade (DRC) model, visual word recognition proceeds via lexical and non-lexical parallel paths (Coltheart et al., 2001). While the DRC model was originally developed to explain reading aloud, its orthographic analysis and lexical decision components provide a vital framework for silent reading as well. Even without vocalization, orthographic nodes in the mental lexicon remain active during silent reading. PWs with low OLD20 values trigger widespread activation of real-word nodes via the lexical route, forcing the system to undergo an additional “monitoring” load to manage the cognitive noise generated by these involuntary activations during the silent reading process.

The contour plots presented in Fig. 3 clearly illustrate the temporal and spatial dynamics of lexicality and orthographic neighborhood effects. In RWs (Panel C), pupil dilation remains within a narrow temporal window, and the impact of OLD20 is relatively limited. In contrast, for PWs (Panel B), pupil dilation is significantly more intense and spreads across a much broader temporal area; furthermore, cognitive load increases as OLD20 values decrease, indicating higher orthographic similarity. The distribution of cognitive load in NWs (Panel A) is weaker than in PWs, as NWs do not resemble existing units in the mental lexicon and therefore do not trigger intense lexical competition. Unlike NWs, the sustained high dilation observed in PWs—particularly at low OLD20 values with high neighborhood density—serves as evidence for the "lateral inhibition" effort exerted by the system to suppress competing lexical candidates.

## Conclusion

This study sheds light on the cognitive processing differences between RWs, PWs, and NWs with varying OLD20 levels in Turkish, a language with transparent orthography. In this context, the RW-PW comparison primarily reveals a lexical/semantic effect triggered by the rich semantic networks of RWs and the intense semantic competition inherent in this process; while RWs achieve a successful match in the mental lexicon, the subsequent semantic integration imposes a deeper cognitive load than the unsuccessful lexical search associated with PWs. Furthermore, the PW-NW comparison highlights the intense orthographic competition created by the high similarity of PWs to real-word forms and the “lateral inhibition”-based inhibitory effort exerted by the system to resolve this competition. Temporally, these two mechanisms exhibit a clear hierarchy: while the lexicality effect driven by semantic activation becomes significant at a relatively early stage, the orthographic neighborhood effect driven by neighborhood density emerges with a distinct temporal lag at a relatively late phase. Consequently, pupillometry is a sensitive method capable of dissociating early cognitive load arising from semantic activation from late-stage load arising from neighborhood competition.

A potential limitation of the current study concerns the assessment of phonotactic legality, which was controlled strictly through position-specific bigram frequency. While this statistical approach effectively captures transitional probabilities between adjacent segments—fundamental to orthographic legality in Turkish—it may not fully reflect the subjective perception of ‘word-likeness’ or syllable-level structural integrity. Specifically, strings that are statistically legal according to bigram counts may still be perceived as orthographically anomalous by native speakers due to higher-order phonotactic violations. Future research could address this by incorporating a behavioral pre-test, such as a ‘word-likeness’ rating task, to empirically validate the phonotactic naturalness of the stimuli.

## Supplementary Information

Below is the link to the electronic supplementary material.

Supplementary file1 (DOCX 1898 kb)

## Data Availability

The data supporting the findings of this study are available from the corresponding author upon reasonable request.
