# Supplementary material for: The Effect of Orthographic Neighbourhood and Semantics on Lexical Processing in a Transparent Orthographic Language: A Pupilometry Study
Source: J Psycholinguist Res. 2026 Jun 13;55(4):83. doi: 10.1007/s10936-026-10267-4 (PMC13264559; doi:10.1007/s10936-026-10267-4)
Supplement: Supplementary file 1 — Supplementary file1 (DOCX 1898 kb) [file 10936_2026_10267_MOESM1_ESM.docx]

**Supplementary Material**

***Power analysis***

The figure below illustrates the outcomes derived from employing the *wp.regression()* function within the *WebPower* package (Version 0.8.7) in R, with power set to 0.80, alpha set to 0.05, two predictors (frequency and word type) evaluated, and f^2^ set to 0.35, for a sample size of 20–50 participants.


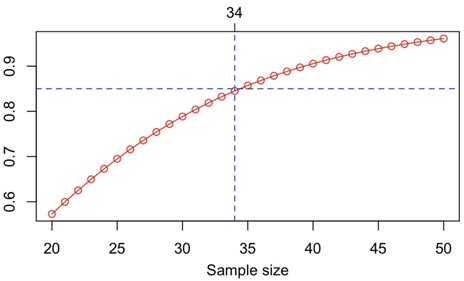


**Figure S1.** Result of power analysis (N=34, f^2^=0.35, alpha=0.05, power= 0.85)

***Material***

**Table S1.** The stimuli used in the experiment and their properties

| N | Word | Trans. | Type | OD | OLD20 | Bigram | Valence | Arousal | Disgust | Concr. | Rank | Freq. | Disp. |
| --- | --- | --- | --- | --- | --- | --- | --- | --- | --- | --- | --- | --- | --- |
| 1 | tnuko | - | NW | high | 3.00 | 5.453 | - | - | - | - | - | - | - |
| 2 | tonuk | - | PW | low | 1.75 | 8.926 | - | - | - | - | - | - | - |
| 3 | konut | housing | RW | low | 1.50 | 9.146 | 5.37 | 3.55 | 40.23 | 83.88 | 1800 | 3.577 | 0.82 |
| 4 | ntsaa | - | NW | high | 2.75 | 7.648 | - | - | - | - | - | - | - |
| 5 | tasna | - | PW | low | 1.80 | 8.843 | - | - | - | - | - | - | - |
| 6 | sanat | art | RW | low | 1.50 | 9.376 | 7.31 | 5.97 | 79.12 | 30.25 | 484 | 20.58 | 0.72 |
| 7 | lmiib | - | NW | high | 3.00 | 5.462 | - | - | - | - | - | - | - |
| 8 | iblim | - | PW | low | 1.80 | 8.303 | - | - | - | - | - | - | - |
| 9 | bilim | science | RW | low | 1.50 | 9.837 | 7.51 | 5.51 | 69.23 | 64.21 | 382 | 21.321 | 0.89 |
| 10 | rdgie | - | NW | high | 3.00 | 6.471 | - | - | - | - | - | - | - |
| 11 | gedir | - | PW | low | 1.55 | 9.239 | - | - | - | - | - | - | - |
| 12 | dergi | magazine | RW | low | 1.55 | 9.693 | 6.17 | 4.29 | 54.09 | 89.26 | 732 | 10.511 | 0.92 |
| 13 | aukkl | - | NW | high | 2.75 | 6.998 | - | - | - | - | - | - | - |
| 14 | kaluk | - | PW | low | 1.70 | 9.546 | - | - | - | - | - | - | - |
| 15 | kulak | ear | RW | low | 1.55 | 9.081 | 5.08 | 4.17 | 24.65 | 83.47 | 730 | 10.757 | 0.89 |
| 16 | aiğtk | - | NW | high | 3.00 | 4.111 | - | - | - | - | - | - | - |
| 17 | katığ | - | PW | low | 1.70 | 9.381 | - | - | - | - | - | - | - |
| 18 | kağıt | paper | RW | low | 1.60 | 9.301 | 4.95 | 3.64 | 41.10 | 89.42 | 1331 | 4.817 | 0.93 |
| 19 | rmsei | - | NW | high | 2.95 | 5.652 | - | - | - | - | - | - | - |
| 20 | sermi | - | PW | low | 1.75 | 9.356 | - | - | - | - | - | - | - |
| 21 | resim | picture | RW | low | 1.65 | 8.483 | 6.78 | 5.39 | 66.58 | 74.48 | 584 | 14.695 | 0.83 |
| 22 | rmied | - | NW | high | 3.00 | 5.903 | - | - | - | - | - | - | - |
| 23 | dermi | - | PW | low | 1.65 | 9.627 | - | - | - | - | - | - | - |
| 24 | demir | iron | RW | low | 1.65 | 9.327 | 5.05 | 4.17 | 25.93 | 81.59 | 1163 | 5.984 | 0.92 |
| 25 | mykee | - | NW | high | 2.95 | 7.472 | - | - | - | - | - | - | - |
| 26 | yemke | - | PW | low | 1.80 | 8.672 | - | - | - | - | - | - | - |
| 27 | yemek | food | RW | low | 1.70 | 8.914 | 7.76 | 6.24 | 81.35 | 85.00 | 369 | 22.103 | 0.90 |
| 28 | ltnaı | - | NW | high | 3.00 | 7.299 | - | - | - | - | - | - | - |
| 29 | lanıt | - | PW | low | 1.75 | 8.616 | - | - | - | - | - | - | - |
| 30 | altın | gold | RW | low | 1.70 | 8.768 | 6.79 | 5.30 | 60.79 | 86.61 | 1034 | 7.242 | 0.91 |
| 31 | rymou | - | NW | high | 3.00 | 5.220 | - | - | - | - | - | - | - |
| 32 | yomur | - | PW | low | 1.70 | 7.913 | - | - | - | - | - | - | - |
| 33 | yorum | comment | RW | low | 1.70 | 8.938 | 4.81 | 3.75 | 24.87 | 51.34 | 913 | 8.319 | 0.91 |
| 34 | znied | - | NW | high | 3.00 | 6.249 | - | - | - | - | - | - | - |
| 35 | dizne | - | PW | low | 1.80 | 8.345 | - | - | - | - | - | - | - |
| 36 | deniz | sea | RW | low | 1.75 | 9.540 | 7.22 | 6.49 | 77.56 | 80.11 | 359 | 22.106 | 0.92 |
| 37 | leakm | - | NW | high | 2.75 | 6.402 | - | - | - | - | - | - | - |
| 38 | mekal | - | PW | low | 1.70 | 9.003 | - | - | - | - | - | - | - |
| 39 | kalem | pen | RW | low | 1.75 | 9.690 | 6.26 | 4.42 | 52.97 | 89.32 | 939 | 7.815 | 0.93 |
| 40 | yraas | - | NW | high | 2.90 | 7.817 | - | - | - | - | - | - | - |
| 41 | asyar | - | PW | low | 1.75 | 8.842 | - | - | - | - | - | - | - |
| 42 | saray | palace | RW | low | 1.75 | 9.818 | 4.51 | 5.00 | 31.43 | 85.86 | 1259 | 5.717 | 0.86 |
| 43 | sneib | - | NW | high | 3.00 | 6.791 | - | - | - | - | - | - | - |
| 44 | sebin | - | PW | low | 1.80 | 8.616 | - | - | - | - | - | - | - |
| 45 | besin | food | RW | low | 1.75 | 8.959 | 6.66 | 4.31 | 55.58 | 83.48 | 1936 | 3.605 | 0.73 |
| 46 | egnsü | - | NW | high | 3.00 | 4.403 | - | - | - | - | - |  | - |
| 47 | güşen | - | PW | low | 1.80 | 8.737 | - | - | - | - | - |  | - |
| 48 | güneş | sun | RW | low | 1.80 | 8.803 | 7.57 | 6.40 | 84.19 | 86.66 | 594 | 13.468 | 0.90 |
| 49 | reekş | - | NW | high | 2.85 | 7.047 | - | - | - | - | - | - | - |
| 50 | eşrek | - | PW | low | 1.80 | 8.752 | - | - | - | - | - | - | - |
| 51 | şeker | sugar | RW | low | 1.80 | 9.004 | 6.50 | 5.10 | 65.39 | 89.41 | 1313 | 5.206 | 0.88 |
| 52 | zhvua | - | NW | high | 3.00 | 5.656 | - | - | - | - | - | - | - |
| 53 | vahuz | - | PW | low | 2.00 | 8.361 | - | - | - | - | - | - | - |
| 54 | havuz | pool | RW | low | 1.80 | 8.628 | 6.36 | 5.23 | 67.06 | 88.48 | 2057 | 2.885 | 0.93 |
| 55 | aitpk | - | NW | high | 2.50 | 7.496 | - | - | - | - | - | - | - |
| 56 | pikat | - | PW | low | 1.85 | 8.391 | - | - | - | - | - | - | - |
| 57 | kitap | book | RW | low | 1.85 | 8.630 | 5.41 | 5.47 | 77.90 | 89.87 | 199 | 40.233 | 0.92 |
| 58 | kçeiç | - | NW | high | 2.85 | 5.501 | - | - | - | - | - | - | - |
| 59 | keçiç | - | PW | low | 1.90 | 8.325 | - | - | - | - | - | - | - |
| 60 | çiçek | flower | RW | low | 1.90 | 8.154 | 5.56 | 4.85 | 76.58 | 90.87 | 675 | 11.94 | 0.88 |
| 61 | mkkee | - | NW | high | 2.95 | 7.412 | - | - | - | - | - | - | - |
| 62 | kemke | - | PW | low | 1.80 | 8.466 | - | - | - | - | - | - | - |
| 63 | ekmek | bread | RW | low | 1.90 | 8.227 | 6.89 | 5.00 | 69.71 | 84.81 | 1090 | 6.576 | 0.91 |
| 64 | rtpia | - | NW | high | 2.95 | 5.655 | - | - | - | - | - | - | - |
| 65 | ripat | - | PW | low | 1.95 | 7.827 | - | - | - | - | - | - | - |
| 66 | parti | party | RW | low | 1.95 | 9.413 | 5.79 | 5.29 | 58.00 | 72.36 | 292 | 31.644 | 0.84 |
| 67 | otrmo | - | NW | high | 2.95 | 7.014 | - | - | - | - | - | - | - |
| 68 | manor | - | PW | low | 1.95 | 8.769 | - | - | - | - | - | - | - |
| 69 | roman | novel | RW | low | 1.95 | 8.674 | 6.73 | 5.35 | 68.32 | 83.53 | 826 | 10.729 | 0.79 |
| 70 | arnmo | - | NW | high | 2.85 | 8.010 | - | - | - | - | - | - | - |
| 71 | torom | - | PW | low | 1.95 | 8.237 | - | - | - | - | - | - | - |
| 72 | motor | motor | RW | low | 1.95 | 7.233 | 5.51 | 4.89 | 32.30 | 88.67 | 1720 | 3.541 | 0.89 |
| 73 | pmkua | - | NW | high | 2.95 | 5.903 | - | - | - | - | - | - | - |
| 74 | mupka | - | PW | low | 1.95 | 7.902 | - | - | - | - | - | - | - |
| 75 | pamuk | cotton | RW | low | 1.95 | 8.488 | 6.54 | 4.70 | 52.18 | 88.76 | 2383 | 2.077 | 0.92 |
| 76 | vkhea | - | NW | high | 2.95 | 4.232 | - | - | - | - | - | - | - |
| 77 | havek | - | PW | low | 1.95 | 8.970 | - | - | - | - | - | - | - |
| 78 | kahve | coffee | RW | low | 1.95 | 9.364 | 6.95 | 6.31 | 66.80 | 93.91 | 1662 | 7.2 | 0.87 |
| 79 | braeh | - | NW | high | 3.00 | 7.480 | - | - | - | - | - | - | - |
| 80 | rebah | - | PW | low | 1.90 | 7.731 | - | - | - | - | - | - | - |
| 81 | haber | news | RW | low | 1.95 | 9.046 | 4.53 | 4.81 | 20.42 | 61.87 | 304 | 28.211 | 0.90 |
| 82 | ükrpö | - | NW | high | 3.00 | 5.368 | - | - | - | - | - | - | - |
| 83 | pökür | - | PW | low | 2.00 | 7.154 | - | - | - | - | - | - | - |
| 84 | köprü | bridge | RW | low | 2.00 | 6.802 | 5.44 | 3.65 | 39.96 | 90.55 | 1521 | 4.058 | 0.91 |
| 85 | crüeh | - | NW | high | 3.00 | 5.065 | - | - | - | - | - | - | - |
| 86 | hücer | - | PW | low | 1.95 | 8.325 | - | - | - | - | - | - | - |
| 87 | hücre | cell | RW | low | 2.00 | 7.618 | 4.49 | 4.03 | 16.80 | 83.34 | 850 | 11.522 | 0.70 |
| 88 | ryoad | - | NW | high | 3.00 | 6.039 | - | - | - | - | - | - | - |
| 89 | yardo | - | PW | low | 1.90 | 9.794 | - | - | - | - | - | - | - |
| 90 | radyo | radio | RW | low | 2.15 | 8.182 | 6.43 | 4.98 | 66.97 | 92.20 | 1184 | 6.24 | 0.85 |

*Note.* N= Number, Trans. = Translation, OD = Orthographic Distance, OLD20 = Orthographic Levenshtein Distance; Disgust. = Disgustness, Concr. = Concreteness, Freq=Frequency; Disp. = Dispersion, PW = pseudoword, NW = nonword, RW = real word, Values for bigram frequencies are scaled by 10^3^. All conditions (RW, PW, NW) consist of the same set of letters. Unique segment count (*N* = 27) and Shannon Diversity Index (*H* = 4.31) are identical across all groups.


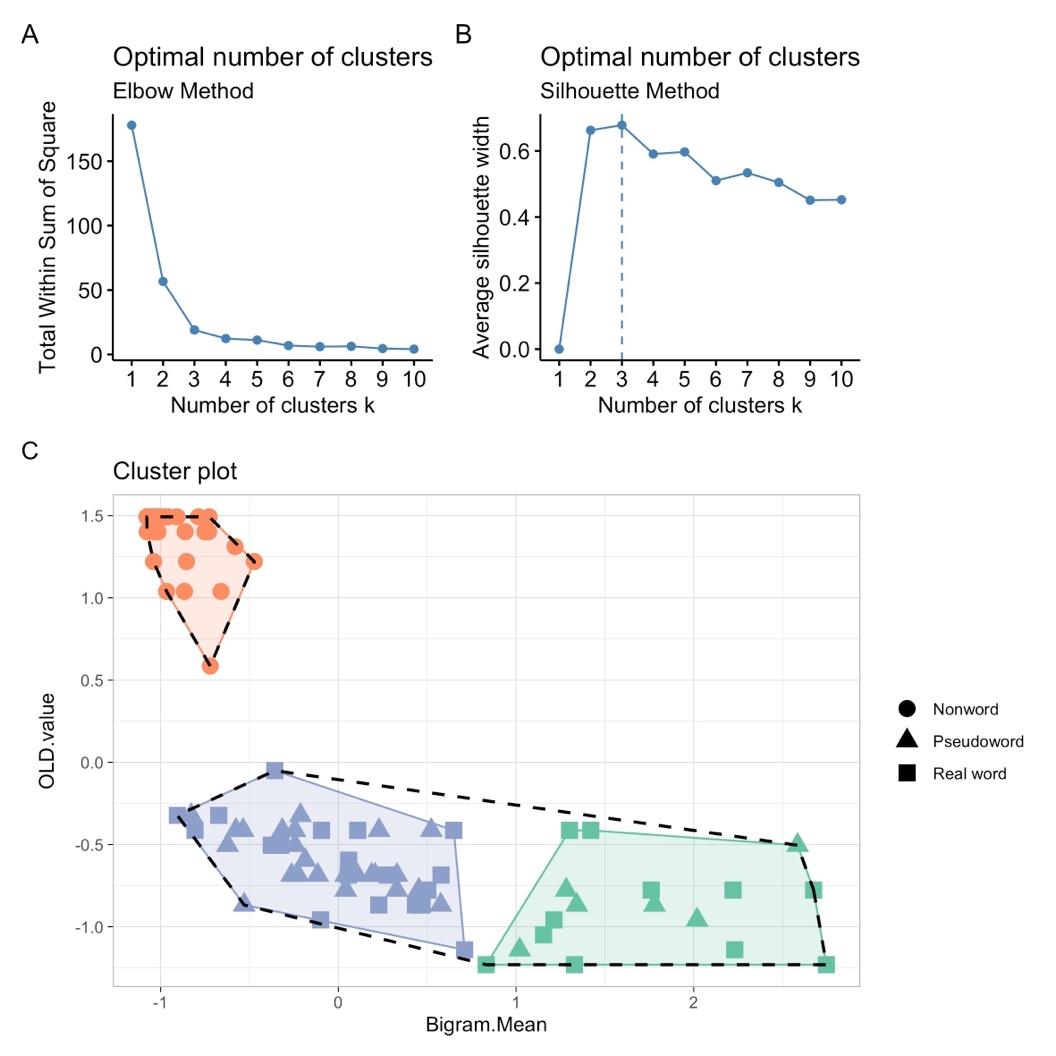


**Figure S2**. Structural validation of the stimulus set via clustering analysis.

To validate the orthographic characteristics and distributional properties of the stimuli (real words, pseudowords, and nonwords), a k-means clustering analysis was conducted. Elbow and Silhouette metrics (Figures A and B) support a three-cluster (k=3) solution as an optimal structure for the dataset. While k=3 was selected for higher granularity, the statistical distinction between k=2 and k=3 remains marginal, as both solutions effectively highlight the primary morphological division. The scaled centroid coordinates are as follows: Cluster 1 (Bigram: 1.70, OLD20: -0.89), Cluster 2 (Bigram: -0.92, OLD20: 1.36), and Cluster 3 (Bigram: -0.03, OLD20: -0.60).

The analysis yielded an Adjusted Rand Index (ARI) of 0.518, indicating a moderate-to-high alignment between statistical clusters and experimental categories. This alignment is primarily driven by the clear isolation of all 30 nonwords within Cluster 2 (high OLD20). In contrast, pseudowords and real words exhibit a significant overlap within the low-OLD20 pool, distributed between Cluster 1 and Cluster 3. Specifically, 24/30 pseudowords and 19/30 real words coalesced in Cluster 3, confirming that these two categories were successfully matched for key structural variables. In Figure C, the dashed black convex hulls illustrate that the dataset fundamentally bifurcates into two macro-groups based on OLD20 (Low vs. High), where low-OLD20 stimuli (real and pseudowords) form a structurally cohesive block distinct from high-OLD20 nonwords.

**Table S2.** Descriptive statistics of orthographic distance (OLD20) and bigram frequency for each stimulus condition

| Condition | OLD20 M (SD) [Range] | Bigram M (SD) [Range] |
| --- | --- | --- |
| Nonword | 2.93 (0.11) [2.50–3.00] | 6.24 (1.10) [4.11–8.01] |
| Pseudoword | 1.82 (0.11) [1.55–2.00] | 8.63 (0.60) [7.15–9.79] |
| Real Word | 1.78 (0.17) [1.50–2.15] | 8.84 (0.73) [6.80–9.84] |

*Note: M*=Mean, *SD*= Standard Deviation

**Table S3.** Results of normality, homogeneity, and group comparison tests for OLD20 and bigram metrics

| Metric | Normality (A) | Homogeneity (F) | Kruskal-Wallis (χ2) | Post-hoc Comparison | Adjusted p (Bonf.) |
| --- | --- | --- | --- | --- | --- |
| OLD20 | 8.50^***^ | 5.28^**^ | 60.36^***^ | NW vs. PW | <.001 |
|  |  |  |  | NW vs. RW | <.001 |
|  |  |  |  | PW vs. RW | 1.000 |
| Bigram | 2.66^***^ | 8.12^***^ | 55.92^***^ | NW vs. PW | <.001 |
|  |  |  |  | NW vs. RW | <.001 |
|  |  |  |  | PW vs. RW | .877 |

*Note:* * *p* <.05, ***p* < .01, *** *p* < .00. NW = Nonword, PW = Pseudoword, RW = Real Word.

***LME results***

In the statistical analysis, a model reduction process was followed where fixed effects were kept constant while only the random effects structure was optimized. Specifically, the Full Model, which included all random slopes, was compared against the Final Model derived via backward elimination based on the AIC (Akaike Information Criterion) using the *buildmer*package. The results (see Table S4) indicate that for all three parameters, the Final Models yielded lower AIC values and fewer degrees of freedom (*df*). This confirms that optimizing the random structure while maintaining constant fixed effects resulted in a more parsimonious model that provides a superior fit to the data.

The LME analysis for static pupillary indices revealed a distinct functional dissociation between orthographic effort and lexical resolution. Mean Pupil Diameter and Peak Pupil Amplitude were significantly predicted by Orthographic Distance (OLD20). Stimuli with higher orthographic similarity (lower OLD20 values) elicited larger pupillary responses (Table S5, Figure S3), confirming that cognitive effort intensity is driven by competition within the orthographic neighborhood. In contrast, Latency of the Peak Pupil Dilation was exclusively modulated by Lexicality (p<.001), with real words (RW) reaching peak dilation significantly earlier than pseudowords (PW). These effects were observed using sum-coded predictors for both Lexicality and Orthographic Distance, ensuring that the results reflect robust main effects across the experimental conditions.

**Table S4.** Comparison of Linear Mixed-Effects (LME) models for pupil metrics.

|  | df | AIC | Model |
| --- | --- | --- | --- |
| *Mean Pupil Diameter* | | | |
| Final Model | 6 | 5655.78 | *Mean ~ 1 + Lex + Orth + Bigram + (1 \| Subject)* |
| Full Model | 17 | 5667.54 |  |
| *Peak Pupil Amplitude* | | | |
| Final Model | 8 | 5899.85 | *Peak ~ 1 + Lex + Orth + Bigram + (1 + Lex \| Subject)* |
| Full Model | 17 | 5908.53 |  |
| *Latency of Peak Dilation* | | | |
| Final Model | 7 | 36179.58 | *Latency ~ 1 + Lex + Orth + Bigram + (1 \| Subject) + (1 \| Item)* |
| Full Model | 17 | 36193.39 |  |

*Note.* The full model structure is specified as follows: ~ 1 + Lex + Orth + Bigram + (1 + Lex + Orth | Subject) + (1 | Item). In the analysis, fixed effects were kept constant across all models, while only the random effects structure was optimized. Abbreviations: Lex: Lexicality, Orth: Orthographic neighborhood, Bigram: Log-transformed bigram frequency.

**Table S5**. Summary of Linear Mixed-Effects (LME) models for mean pupil diameter, peak pupil amplitude, and latency of the peak pupil dilation.

| Predictor | β | SE | t value | Pr(>\|t\|) |
| --- | --- | --- | --- | --- |
| *Mean Pupil Diameter* |  |  |  |  |
| (Intercept) | 0.284 | 0.121 | 2.355 | 0.019 |
| Lexicality | 0.018 | 0.030 | 0.621 | 0.535 |
| Orthographic neighborhood | -0.110 | 0.046 | -2.403 | **0.016** |
| Bigram (log) | 0.002 | 0.015 | 0.136 | 0.892 |
| *Peak Pupil Amplitude* |  |  |  |  |
| (Intercept) | 0.548 | 0.125 | 4.366 | <.001 |
| Lexicality | 0.034 | 0.038 | 0.899 | 0.374 |
| Orthographic neighborhood | -0.110 | 0.048 | -2.306 | **0.021** |
| Bigram (log) | -0.002 | 0.015 | -0.121 | 0.904 |
| *Latency of Peak Dilation* |  |  |  |  |
| (Intercept) | 842.71 | 29.52 | 28.54 | <.001 |
| Lexicality | -28.62 | 7.26 | -3.94 | **<.001** |
| Orthographic neighborhood | -5.64 | 11.17 | -0.51 | 0.615 |
| Bigram (log) | -1.13 | 3.57 | -0.32 | 0.752 |

*Note*: *ß* represents the estimate for parametric coefficients Predictors included Log-transformed Bigram frequency. Since the distribution of OLD20 (Orthographic Levenshtein Distance) values followed a normal distribution, no further normalization was applied. Both Lexicality and Orthographic neighborhood (based on OLD20 categories) were sum-coded to allow for the estimation of main effects.

**
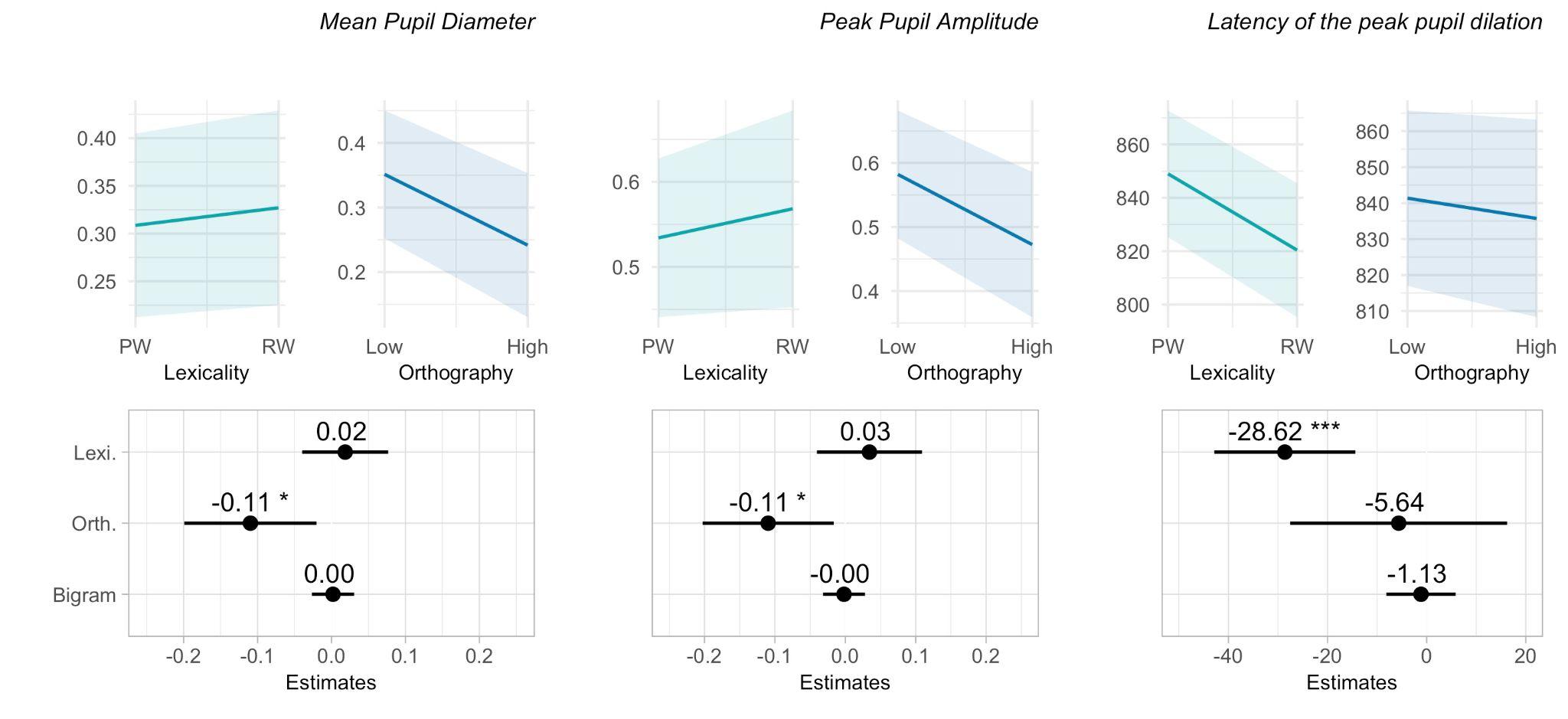
**

**Figure S3.** Visualization of LME results for pupillary static indices. The top row displays the marginal effects (predicted values) for Lexicality and Orthography across Mean Pupil Diameter, Peak Amplitude, and Latency. The bottom row presents the fixed-effect estimates (β) with 95% confidence intervals. Asterisks indicate statistical significance levels: p < .001, ** p < .01, * p < .05.

***GAMM results***

**Table S6**. Summary of the Generalized Additive Mixed Model (GAMM) for pupil dilation

| Predictor | ß/edf | SE/Ref.df | t/F | p |
| --- | --- | --- | --- | --- |
| *Parametric Coefficients* |  |  |  |  |
| (Intercept) | 0.68 | 6.93 | 0.1 | .922 |
| Pseudoword | 11.66 | 9.12 | 1.28 | .201 |
| Real word | 4.18 | 9.41 | 0.44 | .657 |
| *Smooth Terms* |  |  |  |  |
| s(Time):Nonword | 8.95 | 8.99 | 193.87 | <.001 |
| s(Time):Pseudoword | 8.96 | 8.99 | 242.85 | <.001 |
| s(Time):Real word | 8.97 | 8.99 | 291.43 | <.001 |
| te(Time, Bigram Log) | 16.11 | 17.92 | 5.83 | <.001 |
| s(Subject) | 31.16 | 33 | 17.17 | <.001 |
| s(Item) | 75.71 | 86 | 8.57 | <.001 |

*Note.*  *ß* represents the estimate for parametric coefficients, while *edf* (effective degrees of freedom) is reported for smooth terms. *SE* represents the standard error for parametric coefficients, and *Ref.df* (reference degrees of freedom) is reported for smooth terms. *N* = 445,752.

**Table S7**. Summary of the Generalized Additive Mixed Model (GAMM) using a binary difference smooth to compare real words and pseudowords

| Predictor | ß/edf | SE/Ref.df | t/F | p |
| --- | --- | --- | --- | --- |
| *Parametric Coefficients* |  |  |  |  |
| (Intercept) | 6.96 | 3.72 | 1.87 | 0.062 |
| *Smooth Terms* |  |  |  |  |
| s(Time) (*Reference: Pseudowords*) | 8.82 | 8.96 | 58.03 | <.001 |
| s(Time):IsRealWord (*Difference: RW-PW*) | 8.36 | 9.48 | 3.51 | **<.001** |
| te(Time, Bigram Log) | 3.37 | 3.97 | 1.8 | 0.127 |
| s(Subject) | 17.41 | 33 | 1.12 | <.001 |
| s(Item) | 0.05 | 59 | 0 | 0.711 |

*Note.*  *ß*and *SE* are reported for parametric coefficients. For smooth terms, *edf* (effective degrees of freedom) indicates the degree of non-linearity, and *Ref.df*represents the reference degrees of freedom used for hypothesis testing. In this binary smooth specification, *s(Time)* represents the trajectory for the reference category (Pseudowords), and *s(Time):IsRealWord* represents the difference in the time course between Real Words and Pseudowords. *N* = 297,168.

**Table S8**. Summary of the Generalized Additive Mixed Model (GAMM) using a binary difference smooth to compare nonwords and pseudowords

| Predictor | ß/edf | SE/Ref.df | t/F | p |
| --- | --- | --- | --- | --- |
| *Parametric Coefficients* |  |  |  |  |
| (Intercept) | 1.28 | 4.45 | 0.29 | 0.774 |
| *Smooth Terms* |  |  |  |  |
| s(Time) (*Reference: Pseudowords*) | 8.85 | 8.98 | 80.9 | <.001 |
| s(Time): IsHighOLD (*Difference: NW-PW*) | 5.23 | 6.58 | 2.34 | **0.034** |
| te(Time, Bigram Log) | 2.02 | 2.03 | 3.68 | 0.025 |
| s(Subject) | 17.77 | 34 | 1.14 | <.001 |
| s(Item) | 0.09 | 60 | 0 | 0.59 |

*Note.*  *ß*and *SE* are reported for parametric coefficients. For smooth terms, *edf*(effective degrees of freedom) indicates the degree of non-linearity, and *Ref.df* represents the reference degrees of freedom used for hypothesis testing. In this binary smooth specification, *s(Time)* represents the trajectory for the reference category (Pseudowords), and *s(Time):IsHighOLD* represents the difference in the time course between Nonwords and Pseudowords. *N* = 297,319.

**Table S9**: GAMM Results for the tensor product of time and orthographic distance (OLD20)

| Predictor | ß/edf | SE/Ref.df | t/F | p |
| --- | --- | --- | --- | --- |
| Parametric Coefficients |  |  |  |  |
| (Intercept) | -26.45 | 65.13 | -0.406 | 0.685 |
| Nonword | 10.44 | 84.2 | 0.124 | 0.901 |
| Real word | 34.64 | 68.41 | 0.506 | 0.613 |
| Tensor Products |  |  |  |  |
| te(Time, OLD value):Nonword | 11.7 | 12.65 | 3.717 | < .001 |
| te(Time, OLD value):Pseudoword | 8.81 | 10.07 | 5.125 | < .001 |
| te(Time, OLD value):Real word | 13.96 | 15.36 | 3.806 | < .001 |
| te(Time, Bigram Log) | 20.82 | 21.73 | 17.378 | < .001 |
| s(Subject) | 31.14 | 33 | 16.965 | < .001 |
| s(Item) | 72.64 | 83 | 8.359 | < .001 |

*Note.*  *ß* and *SE* are reported for parametric coefficients. For smooth terms, *edf* (effective degrees of freedom) indicates the degree of non-linearity, and *Ref.df* represents the reference degrees of freedom used for hypothesis testing.The table displays the two-dimensional tensor product interaction (*te*) between Time and orthographic distance (OLD20) for all three conditions (RW, PW, and NW). High significance levels (*p*<.001) in the smooth terms indicate that the effect of orthographic similarity on pupil dilation is non-linear and changes dynamically over time. Bigram frequency and random effects for Subject and Item were statistically controlled. *N*=445,752.
